# The Impact of Gabapentinoids on Pain-Related Outcomes after Knee and Hip Surgery: A Systematic Review with Meta-Analysis of Randomized Controlled Trials

**DOI:** 10.3390/jcm13144205

**Published:** 2024-07-18

**Authors:** Dmitriy Viderman, Mina Aubakirova, Azamat Salamat, Dastan Kaldybayev, Nurzhamal Sadir, Ramil Tankacheyev, Yerkin G. Abdildin

**Affiliations:** 1 Department of Surgery, School of Medicine, Nazarbayev University, Astana 020000, Kazakhstan; mina.aubakirova@nu.edu.kz (M.A.);; 2 Department of Anesthesiology and Intensive Care, National Research Oncology Center, Astana 010000, Kazakhstan; 3 Department of Mechanical and Aerospace Engineering, School of Engineering and Digital Sciences, Nazarbayev University, Astana 010000, Kazakhstanyerkin.abdildin@nu.edu.kz (Y.G.A.); 4 Department of Spinal Surgery, National Research Neurosurgery Center, Astana 010000, Kazakhstan

**Keywords:** pregabalin, gabapentin, gabapentinoids, knee surgery, hip surgery, acute pain

## Abstract

**Background:** Postoperative pain remains a significant challenge after knee and hip surgeries, two of the most frequently performed procedures, preventing patients from seeking timely surgical help. Gabapentinoids, gabapentin, and pregabalin, have been gaining attention in postoperative pain management. **Methods:** We conducted a meta-analysis to evaluate the efficacy of gabapentinoids in pain management after knee and hip surgery. PubMed, Scopus, and Cochrane Library were searched for relevant randomized controlled trials (RCTs) published before January 2023. **Results:** Fifteen articles reporting 1320 patients were analyzed. Cumulative pain intensity at rest and on movement was lower in the experimental group with the mean difference (MD) = −0.30 [−0.55,−0.05], *p*-value = 0.02, and MD = −0.41 [−0.68,−0.13], *p*-value = 0.004, respectively. However, the difference was not clinically meaningful and lacked statistical significance at each time period. The gabapentinoid group required less opioid consumption in morphine equivalents (MD = −6.42 [−9.07, −3.78] mg, *p*-value < 0.001). There was a lower incidence of postoperative nausea in the experimental group with a risk ratio (RR) of 0.69 [0.55, 0.86], *p*-value < 0.001. A subgroup analysis showed that gabapentinoids reduced pain on movement on postoperative day two after total knee arthroplasty but not hip arthroplasty. There was insufficient data to examine the efficacy of gabapentinoids in the reduction of chronic postoperative pain in knee/hip surgery. **Conclusions:** Thus, gabapentinoids were associated with a reduction in postoperative pain intensity at rest and on movement, morphine consumption, and the incidence of postoperative nausea in the early postoperative period following knee and hip surgeries. However, pain reduction was not clinically relevant. Sedation has not been evaluated in this work and, if performed, this may have influenced the conclusions. An important limitation of this study is that different gabapentinoids, their administration times and dosages, as well as varying intraoperative management protocols, were pooled together.

## 1. Introduction

Knee and hip surgeries are one of the most frequently performed operating room procedures in clinical practice [1]. However, postoperative pain remains a significant challenge after both surgeries. Postsurgical pain is one of the major reasons patients are unwilling to undergo total hip arthroplasty (THA) or total knee arthroplasty (TKA) [2]. During joint replacement surgeries, tissue damage and postoperative inflammation serve as noxious stimuli, detected by nociceptors in the peripheral nervous system, which are transmitted to the spinal cord and then to the central nervous system through action potentials [3]. Additionally, postoperative inflammation leads to the release of inflammatory substances and cytokines, intensifying the pain experience [3]. Eventually, acute pain may potentially evolve into chronic pain [4].

General anesthesia and systemic opioids, while commonly used, cannot completely prevent central sensitization [5]. Moreover, opioids are associated with multiple complications and adverse effects [6,7]. Regional anesthetic methods provide partial relief but come with limitations [8]. Therefore, gabapentinoids, gabapentin, and pregabalin have been gaining attention in postoperative pain management as they inhibit neuronal excitation in the central nervous system, reduce hyperexcitation of dorsal horn neurons, and release excitatory neurotransmitters [9]. Moreover, the side effects of gabapentin are usually mild [9].

Kremer and colleagues summarized the analgesic mechanisms of gabapentinoids [10]. Although gabapentinoids are structurally related to GABA, their analgesic effects are primarily a result of binding to the α2δ subunit of voltage-dependent calcium channels. This binding reduces calcium influx into neurons, decreasing excitatory neurotransmitter release and dampening neuronal hyperexcitability, which is crucial in neuropathic pain. It is suggested that neuropathic pain often involves an increased expression of the α2δ subunit in the dorsal root ganglia and dorsal horn of the spinal cord. However, this increase is not consistent across all neuropathic conditions. Gabapentin and pregabalin normalize these elevated α2δ levels caused by nerve damage, likely by inhibiting their trafficking to presynaptic terminals rather than altering their overall expression. Furthermore, gabapentinoids influence central sensitization by reducing the excitability of dorsal horn neurons and affecting supraspinal regions involved in pain processing. They modulate neuroimmune responses by reducing pro-inflammatory cytokine expression and microglial activation. Gabapentinoids also appear to reverse central hypersensitivity and suppress the hyperactivity of neurons in brain regions associated with pain. Thus, these mechanisms collectively contribute to gabapentinoids’ effectiveness in managing neuropathic pain, despite not directly interacting with GABA receptors.

Pregabalin and gabapentin have been extensively studied for their antinociceptive effects; however, their efficacy in preventing or reducing acute and chronic postoperative pain remains debatable [11]. Although various systematic reviews attempted to establish their analgesic effects in hip and knee surgeries [12,13,14], these have produced varying results.

Therefore, the current meta-analysis aimed to synthesize existing studies to evaluate the efficacy of gabapentinoids for pain management in knee and hip surgery. Specifically, we aimed to assess gabapentinoids’ effect on pain scores, opioid consumption, postoperative nausea and vomiting, and, potentially, on chronic postoperative pain.

## 2. Materials and Methods

### 2.1. Protocol

We developed the protocol for this meta-analysis for relevant articles. The protocol and methods were arranged and approved by all authors. It is publicly available at https://doi.org/10.17605/OSF.IO/SJ92M (accessed on 10 July 2024). We used the “Preferred Reporting Items for Systematic Reviews and Meta-Analyses (PRISMA)” guidelines [15].

### 2.2. Search Strategy and Criteria

**Figure 1:**
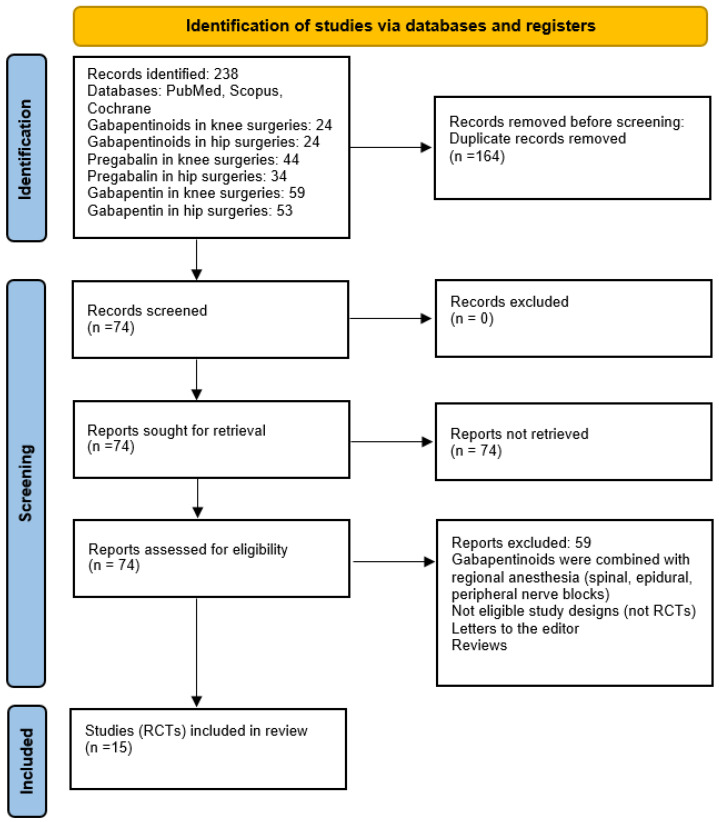
PRISMA diagram. The study selection process.

We searched for RCTs, which studied the analgesic effects of gabapentinoids in the adult population. We searched for relevant articles in the standard databases, such as PubMed and Scopus, as well as the Cochrane Library, published before January 2023 (Figure 1). The following search terms or their combination were used during the search: “pregabalin”, “gabapentin”, “gabapentinoids”, “knee surgery”, “knee arthroplasty”, “knee replacement”, “arthroscopy”, “hip surgery”, “hip replacement”, “hip arthroplasty”, “acute pain”, and “chronic pain”. Two authors independently screened the articles. In case of disagreements, a third author was consulted.

### 2.3. Screening

Screening of the articles was conducted by two authors in an independent manner. In case of disagreements, a third author was consulted. The studies were screened based on titles, then abstracts, and finally, by full texts.

#### 2.3.1. Inclusion Criteria

The inclusion criteria were as follows:

- Patients: Patients aged 18 years old and older undergoing knee or hip surgery (knee arthroplasty, total knee replacement, ligament repair; hip arthroplasty, hip replacement);
- Intervention: Analgesic use of gabapentinoids (pregabalin or gabapentin);
- Control: Placebo;
- Outcomes: Primary—acute postoperative pain intensity at rest and on movement; secondary—postoperative morphine consumption (mg), postoperative nausea and vomiting, chronic postoperative pain;
- Study design: Randomized controlled trials (RCTs).

#### 2.3.2. Exclusion Criteria

The exclusion criteria were as follows:

- Pediatric studies;
- Other comparators;
- Non-RCTs: retrospective studies, case reports, case series, editorials, cadaver studies, and technical reports;
- Not properly described study methodology, assessment, and/or reporting methods;
- Inability to retract the full text.

### 2.4. Assessment of Methodologic Quality

Two authors (MA and NS) independently appraised the quality of each study using the Cochrane risk of bias 2 tool [16] and each outcome using GRADE [17]. Discrepancies were resolved by discussion until reaching a consensus or with the involvement of a third author (DV) if required. The Cochrane risk of bias 2 tool assessed studies as having “low risk”, “some concerns” or “high risk” of bias based on the “randomization process”, “deviations from intended interventions”, “missing outcome”, “measurement of the outcome data”, and “selection of the reported results”. An overall risk of bias was then determined based on these five domains. A GRADE evaluation was performed for the main cumulative outcomes. Each was assessed based on the “risk of bias”, “inconsistency”, “imprecision”, and “indirectness”. Based on these, the outcomes were downgraded or upgraded to a “high”, “moderate”, “low”, or “very low” certainty of evidence.

### 2.5. Data Extraction and Statistical Methods

We extracted and entered qualitative data describing the studies in a data table. Specifically, the following rubrics were used: study reference, 1st author followed by year of publication and country, design, goals (objectives) of the study, age of participants, surgery type, sample size, physical status of patients based on “American Society of Anesthesiologists” (ASA), pharmacological agents (analgesics, hypnotics, adjuvants), and observed side effects. Numeric data for statistical analysis were extracted in a spreadsheet. Some missing statistics were calculated using the methods developed by Luo et al. [18] and by Wan et al. [19]. The meta-analysis was conducted in the “Review Manager software (RevMan, version 5.4)”. Since the studies reported values from different populations, we used the random effects model. The mean difference or risk ratio was used for the examination of the effect size. Forest plots were built for each outcome. Statistical heterogeneity was estimated by the I^2^ statistic.

## 3. Results

### 3.1. Included Studies

The systematic search retrieved 238 original articles (Figure 1). After duplicate removal, 74 articles were screened and 15 articles [9,20,21,22,23,24,25,26,27,28,29,30,31,32] comprising 1320 patients (gabapentinoids—659, controls—661) matched the criteria and were analyzed (Figure 1, Table 1).

### 3.2. Pain Intensity at Rest (0–10 Scale)

**Figure 2:**
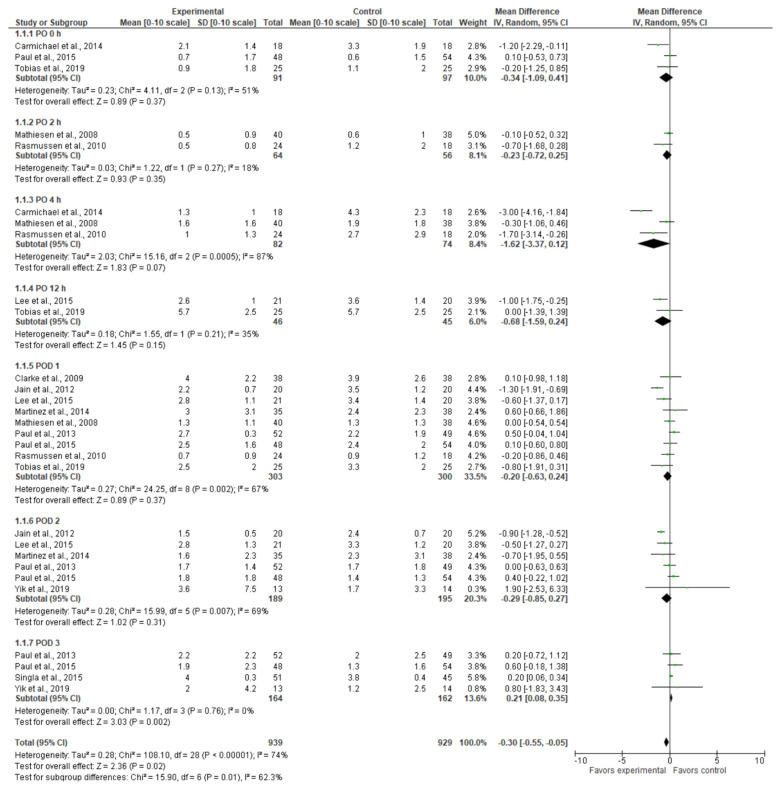
Pain intensity at rest [5,9,20,21,22,23,24,25,26,27,28,29].

The pain intensity score at rest on the Numerical Rating Scale (NRS) and Visual Analog Scale (VAS) is presented in a forest plot in Figure 2. As can be seen from the forest plots, the experimental group tended to have lower pain scores at almost all the time periods, although the difference in pain scores was not statistically significant. However, on POD 3, the experimental group had higher pain scores than the controls (MD = 0.21 [0.08, 0.35], *p* = 0.002). All of the time periods pooled together show an overall effect favoring the gabapentinoid group (MD = −0.30 [−0.55,−0.05], *p*-value = 0.02). This difference is not clinically meaningful. The result is sensitive to the exclusion of some studies (e.g., Carmichael et al., 2019 [21]). The model shows moderate heterogeneity (I^2^ = 74%).

### 3.3. Pain Intensity Score on Movement (0–10 Scale)

**Figure 3:**
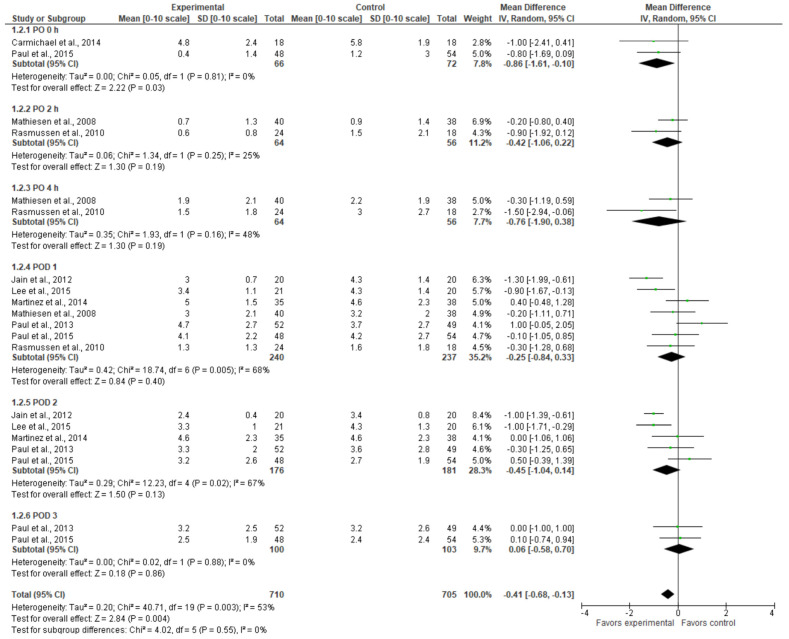
Pain intensity score on movement [9,20,21,22,23,24,25,26,27].

Similar to pain at rest, pain on movement tended to be lower in the experimental group up to day three (Figure 3). Immediately post-surgery, the gabapentinoid group had significantly lower pain scores (MD = −0.86 [−1.61, −0.10], *p* = 0.03). After that, the difference in pain scores was not statistically significant between the two groups. When pooled together, the cumulative pain at all the time periods was lower in the experimental group (MD = −0.41 [−0.68, −0.13], *p*-value = 0.004). The result is not sensitive to the exclusion of any study. The model shows moderate heterogeneity (I^2^ = 53%).

### 3.4. Postoperative Opioid Consumption in Morphine Equivalents (mg)

**Figure 4:**
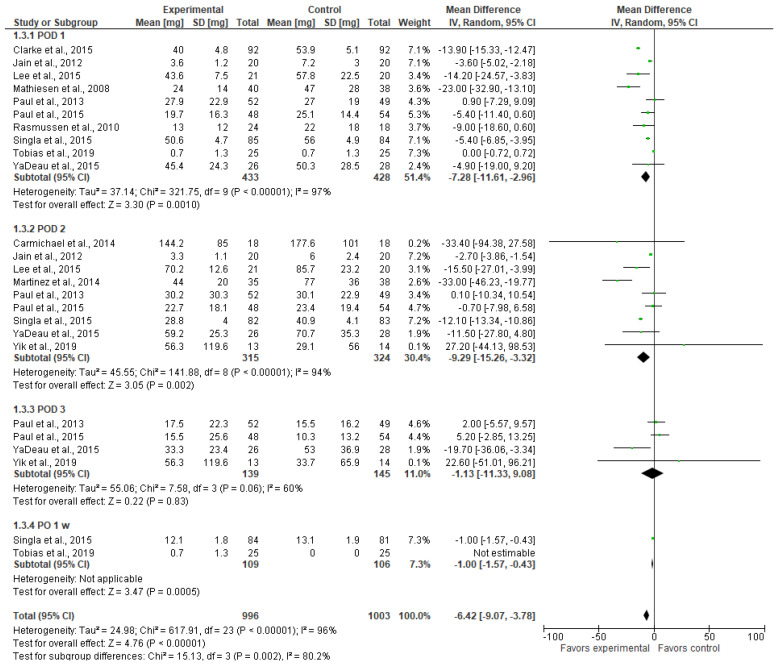
Postoperative opioid consumption in morphine equivalents (mg) [9,20,21,22,23,24,25,26,27,28,29,30,32].

Many studies reported opioid consumption in morphine equivalents in mg (Figure 4). Since fentanyl is almost 100 times more potent than morphine, we converted the fentanyl consumption reported in Lee et al. 2015 [9] in µg into morphine equivalents by dividing the fentanyl values (µg) by 1000 to have in mg and then multiplying by 100. We should also note that Lee et al. 2015 [9] reported tramadol consumption as a rescue medication, so we have not counted it as an opioid in morphine equivalents. Tramadol consumption in two groups “showed no significant difference” [9]. Tobias et al. 2019 [23] reported the consumption of morphine, tramadol, and ketoprofen. However, the latter two were not counted in our report because ultimately “intravenous morphine was administered until pain control” [23].

Opioid consumption was lower in the gabapentinoid group on POD 1 (MD = −7.28 [−11.61, −2.96], *p* = 0.001), POD 2 (MD = −9.29 [−15.26, −3.32], *p* = 0.002), and week 1 (MD = −1.00 [−1.57, −0.43], *p* < 0.001). The overall result of the model favors the gabapentinoid group (MD = −6.42 [−9.07, −3.78], *p* < 0.001). The result is insensitive to the exclusion of any study. The model shows considerable heterogeneity (I^2^ = 96%).

### 3.5. Postoperative Nausea (n)

**Figure 5:**
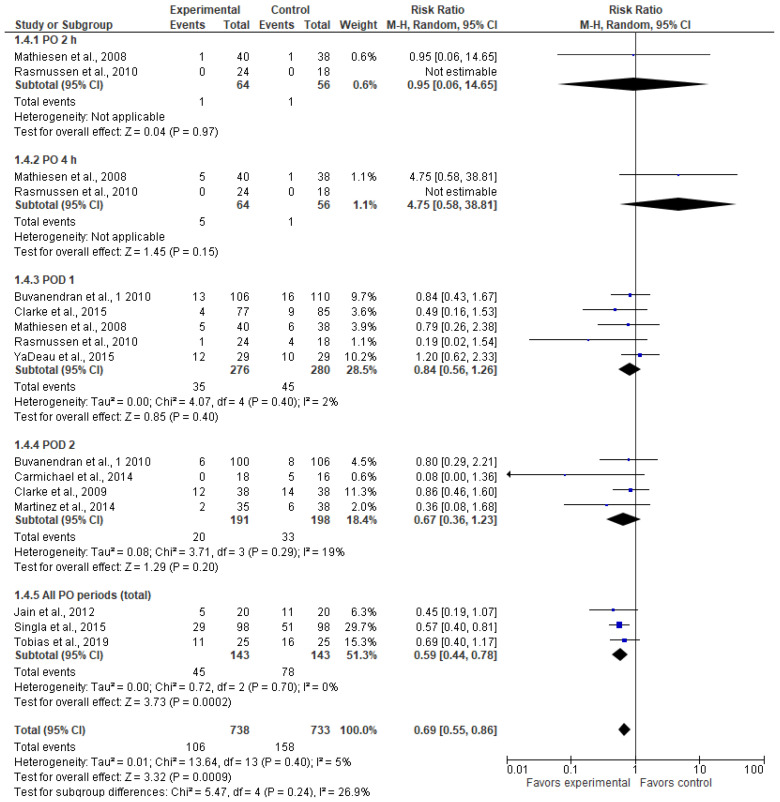
Postoperative nausea (n) [5,23,24,25,26,27,29,30,31,32].

The incidence of nausea was comparable between the two groups at two and four hours after surgery and on postoperative days 1 and 2 (Figure 5). The incidence of nausea was lower in the experimental group in the “all PO periods” subgroup. In this subgroup, Tobias et al., 2020 [23] reported data values measured two months after surgery, while Jain et al., 2012 [26] and Singla et al., 2015 [29] reported data values for all study periods (i.e., total numbers). Combining all these periods shows a lower overall incidence of nausea in the gabapentinoid group (RR = 0.69 [0.55, 0.86], *p*-value = 0.0009, I^2^ = 5%).

### 3.6. Postoperative Vomiting (n)

**Figure 6:**
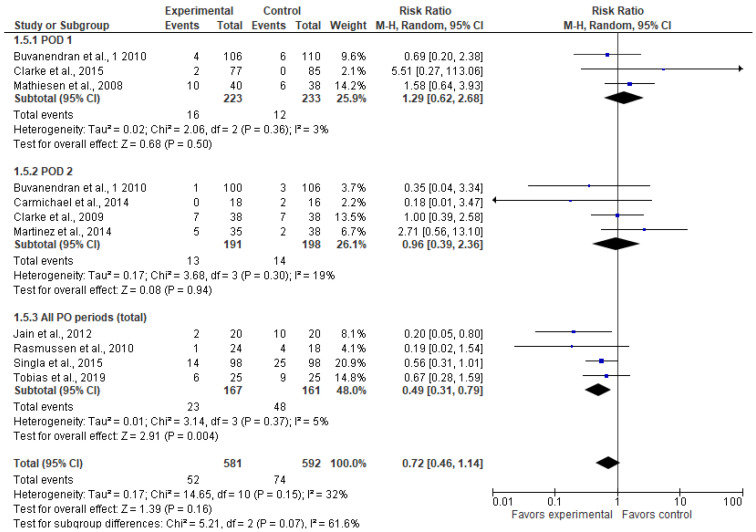
Postoperative vomiting (n) [5,21,23,24,25,26,27,29,31,32].

The incidence of postoperative vomiting on POD 1 and 2 was comparable between the two groups (Figure 6). In the “all PO periods” subgroup, Tobias et al., 2020 [23] reported data values measured two months after surgery, while Jain et al., 2012 [26], Rasmussen et al., 2010 [25], and Singla et al., 2015 [29] reported data values for all study periods (i.e., total numbers). In this subgroup, the gabapentinoid arm had a lower incidence of vomiting (RR = 0.49 [0.31, 0.79, *p* = 0.004, I^2^ = 5%). Overall, the model does not favor the experimental group over the control group (RR = 0.72 [0.46, 1.14], *p*-value = 0.16, I^2^ = 32%).

### 3.7. Subgroup Analysis for TKA and THA: Pain at Rest

**Figure 7:**
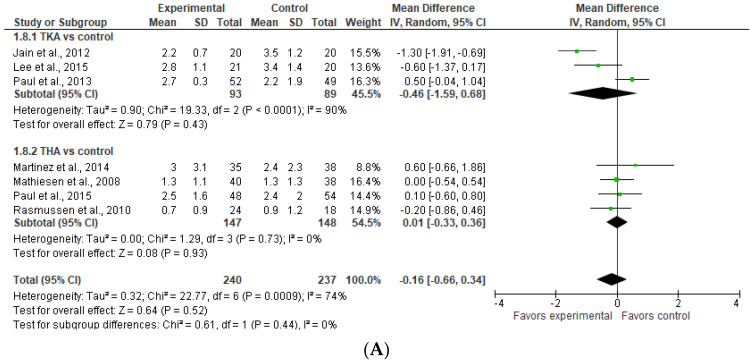

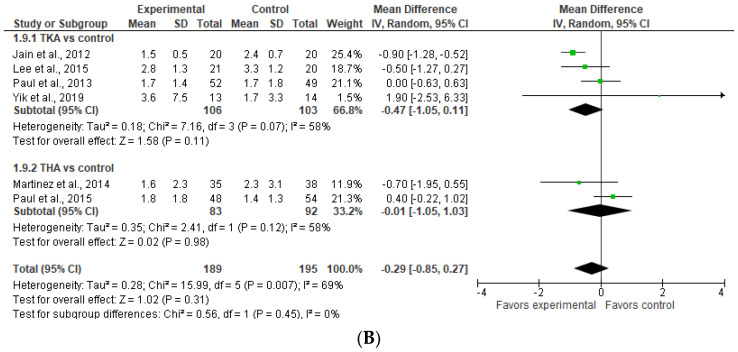
Postoperative pain at rest for THA and TKA on (**A**) POD 1 (upper) and (**B**) POD 2 (lower) [9,20,22,26,27,28].

As evident from Figure 7A (upper part), there is no significant difference between TKA and THA versus the control in pain intensity at rest on POD 1. The result of TKA vs. the control is sensitive to the exclusion of a study by Paul et al. (2013) [20], in which case the model would favor TKA over the control. If we had used the fixed effect model, it would have favored TKA over the control. Similar results were observed on POD 2 (Figure 7B).

### 3.8. Subgroup Analysis for TKA and THA: Pain on Movement

**Figure 8:**
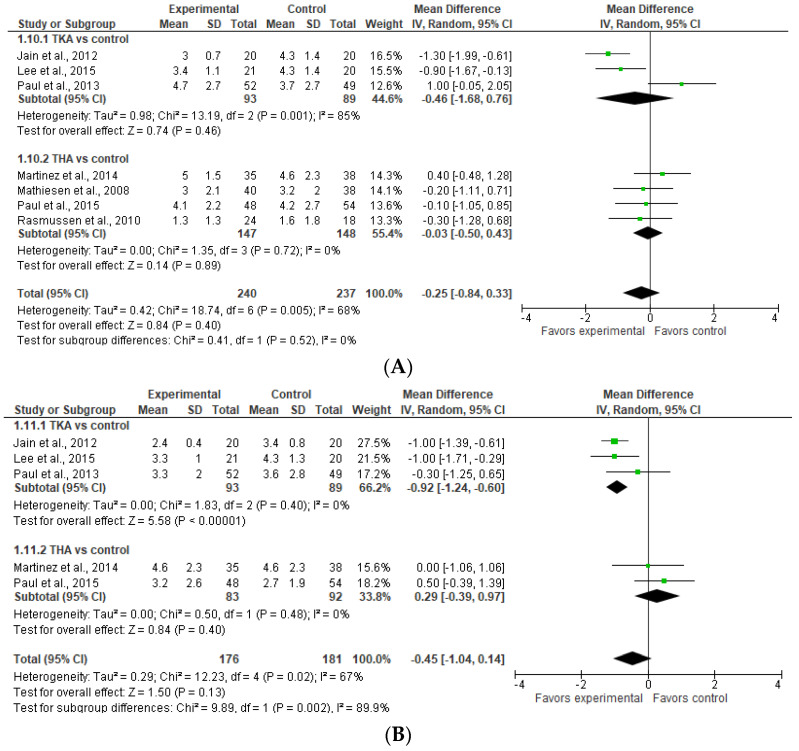
Postoperative pain on movement for THA and TKA on (**A**) POD 1 (upper) and (**B**) POD 2 (lower) [9,20,22,24,25,26,27].

As shown in Figure 8A (upper part), there is no significant difference in pain on movement between TKA and THA versus the control. The result of TKA vs. the control is sensitive to the exclusion of a study by Paul et al. (2013) [20], in which case the model would favor TKA over the control. If we had used the fixed effect model, it would have favored TKA over the control.

### 3.9. Quality Assessment

The results of the Cochrane risk of bias tool 2 assessment are presented in Table 2. Seven studies had a “low risk” of bias, and eight studies had “some concerns” with regard to the risk of bias.

The results of the GRADE assessment of the main outcomes are presented in Table 3. Two outcomes had “high” and three outcomes had a “moderate” level of certainty of evidence.

## 4. Discussion

### 4.1. Interpretations of Results

#### 4.1.1. Pain

In this meta-analysis, pain scores at rest and on movement were lower for the gabapentinoid group from the first postoperative hours up to day three. Although this difference was not statistically significant across most time periods, the analysis may have lacked the power to reach significance due to the small number of participants. Gabapentinoids may provide effective pain management in the first hours post-surgery, and this effect declines by day three. In fact, in our analysis, pain scores at rest on day three were statistically significantly higher for the experimental group, even though the effect size was negligible. Potential contributing factors to such a trend may be the pharmacokinetics and temporal profiles of gabapentinoids, the role of central sensitization, the combination of gabapentinoids with other analgesic agents, or the “rebound pain” effect.

The previous literature has demonstrated varying findings. Similar to our results, pain at rest on days one and two, but not three was found to be lower in the pregabalin group following THA and TKA [33]. Likewise, a meta-analysis of 322 clinical trials comprising all types of surgeries also found the preoperative administration of gabapentinoids effective in alleviating postoperative pain at six, twelve, twenty-four, and forty-eight but not at seventy-two hours post-surgery [34]. It is worth noting that in their subgroup analysis, this remained true for gabapentin, while for pregabalin, the difference in pain intensity lost statistical significance between the two groups at 48 h. This may be associated with pregabalin’s faster absorption: its maximum concentration in the serum is one hour, as opposed to two to four hours for gabapentin [35]. On the other hand, in a meta-analysis of gabapentin use in TKA patients, Han et al. did not find any difference in pain scores at 12, 24, or 48 h [11]. Similarly, Mao et al. did not find differences in pain scores at rest and on movement at 24 and 48 h following THA [36].

In our study, the reduction in pain scores was not clinically meaningful as defined by previous research [37]. Verret et. al. mention that in their study, the pain-sparing effect was more evident in the first hours following the surgery [34]. This again suggests that gabapentinoids are more active in the first postoperative hours. Interestingly, their subgroup analysis showed that the mean difference between the gabapentinoid and control groups for almost all the periods was higher for the gabapentin group rather than pregabalin [34], although the latter is considered to be more potent [35].

#### 4.1.2. Opioid Consumption

Similar to pain intensity, our results showed that there was a significant difference in opioid use on the first two postoperative days, while on day three, the use of opioids was already comparable between the two groups. Lower opioid consumption in the first two days following TKA was observed between the gabapentin and control groups [11] and between the gabapentinoids and control groups following lower limb arthroplasty [13]. Both gabapentin and pregabalin have been found to be effective in reducing opioid use on the first and second postoperative days after THA [36]. Pregabalin substantially decreased morphine consumption following both THA and TKA for 48 h post-surgery [33]. The large meta-analysis comprising various surgeries by Verret et al. found lower cumulative opioid consumption in the gabapentinoid group on postoperative days one (117 trials), two (24 trials), and, unlike our study, three (four trials), compared to controls [34]. One explanation for this opioid-sparing observation may be attributed to gabapentinoids’ ability to strengthen opioids’ effect when taken concurrently [35]. In other words, it might be that fewer opioids were consumed in the experimental group because their effect became more substantial in the presence of gabapentinoids. Another meta-analysis observed a lowered use of opioids for three days following both the knee and hip arthroplasty between the pregabalin and placebo groups but no difference in opioid use between the gabapentin and placebo arms [14]. This, again, may be attributed to pregabalin’s higher potency and, potentially, a higher synergistic effect on opioids.

#### 4.1.3. Postoperative Nausea and Vomiting

We found the cumulative all-period incidence of nausea, but not vomiting, to be lower in the gabapentinoid group. This is consistent with the observed lower consumption of opioids, a factor often associated with postoperative nausea. However, the reduction in opioid consumption was more significant than that of the incidence of nausea, which poses the question of whether the latter was a result of the former. Indeed, Verret et al. found no association between a lower incidence of postoperative nausea and vomiting in the gabapentinoid group with morphine consumption [34]. A lower incidence of nausea, but not vomiting, was observed in the gabapentin group following THA [12]. Han et al. observed a comparable incidence of nausea between the gabapentin and placebo groups following lower limb arthroplasty [11]. Hannon et al. also observed no difference in the incidence of nausea between the gabapentin and placebo groups following TKA; however, their analysis did show a lower incidence of nausea in the pregabalin group compared to the placebo [14]. Likewise, pregabalin was shown effective in lowering the incidence of nausea in TKA but not THA [33].

#### 4.1.4. Chronic Pain

We initially aimed to examine the impact of gabapentinoids on chronic postoperative pain. However, we lacked sufficient data to investigate this relationship in our meta-analysis. This aspect is crucial because while acute pain can be managed with various medications and nerve blocks, there are limited options proven to prevent or reduce chronic pain after surgery. Previously, in a meta-analysis of almost 3200 patients undergoing various types of surgeries, no association was found between preoperative administration of gabapentinoids with chronic pain within three to twelve months [34]. This result held across different gabapentin/pregabalin doses and administration methods. Exploring gabapentinoids’ specific effects on chronic pain following knee surgery might require a longer usage period for their anti-inflammatory effects to become evident. Moreover, the relationship between neuropathic pain in acute settings and the development of chronic postsurgical or neuropathic pain is still not fully understood. Sensitization in central or peripheral nerves is complex and involves changes in how nerves function. Therefore, while gabapentinoids might offer pain relief to some extent, it is important to manage acute postoperative pain using multimodal analgesia.

### 4.2. Study Limitations

Our study has several limitations. First, relatively small RCTs were included in the meta-analysis, and since these reported the outcomes of interest at various times, our study had a small sample size for the majority of the time periods. Second, including studies using either pregabalin or gabapentin and in varying dosages introduced variability in the results, potentially impacting the overall findings. Third, the absence of standardization in surgical procedures across the studies may have contributed to varying pain experiences, making it challenging to draw universal conclusions. Moreover, the demographic variations among patients, including age, gender, and underlying health conditions, might have influenced individual responses to gabapentinoids, further complicating the interpretation of the results. Finally, the lack of long-term follow-up limited our understanding of the sustained effects and potential long-term side effects of gabapentinoids in postoperative pain management.

### 4.3. Implications for Research and Practice

Given what is mentioned above, large and high-quality randomized controlled trials on the topic should be conducted to be able to draw more definitive conclusions. Furthermore, exploring the neurobiological mechanisms, including central sensitization modulation and neuroinflammatory processes, can deepen our understanding of gabapentinoids’ analgesic actions and the temporal effects, potentially leading to the development of more targeted interventions. The synergistic effects of gabapentinoids in combination with other analgesic agents, both opioid and non-opioid, should be studied in-depth to optimize multimodal pain management strategies. Future studies should also focus on examining the effect of gabapentinoid use, especially in conjunction with opioids, on postoperative nausea and vomiting. Further research should also explore the long-term effects of gabapentinoids on postoperative pain management, especially focusing on chronic pain development following knee surgeries. Longitudinal studies with extended follow-up periods are essential to assess the persistence of gabapentinoids’ effects and their role in preventing chronic postoperative pain. As for practical implications, while our results have little clinical importance on their own, they might be useful for developing effective multimodal analgesic interventions for knee and hip surgical procedures.

## 5. Conclusions

This meta-analysis demonstrated that gabapentinoids were associated with reductions in postoperative pain at rest and with movement (however, the reduction was not clinically relevant), morphine consumption, and incidence of postoperative nausea over two postoperative days when knee and hip surgery were combined in the same model. The subgroup analysis showed that gabapentinoids reduced pain on movement on postoperative day two after total knee arthroplasty but not after total hip arthroplasty. Pain reduction was not clinically relevant. Sedation has not been evaluated in this work and, if performed, this may have influenced the conclusions. There was no significant difference between the groups in terms of vomiting during the early postoperative period. There was an insufficient amount of data to support the efficacy of gabapentinoids in the prevention or reduction of chronic postoperative pain in knee and hip surgery. An important limitation of this study is that different gabapentinoids, their administration times and dosages, as well as varying intraoperative management protocols, were pooled together. Having standardized protocols would facilitate further investigation of this issue. Future large, high-quality RCTs are warranted to study the role of gabapentoids in orthopedic surgery, with a focus on the incidence of chronic postoperative pain.

## Figures and Tables

**Table 1:** Characteristics of the included studies. Abbreviations: A, analyzed; ASA, American Society of Anesthesiologists; N, number; NRS, Numeric Rating Scale; PCA, patient-controlled analgesia; PONV, postoperative nausea and vomiting; R, randomized; RCTs, randomized controlled trials; ROM, range of motion; TKA, total knee arthroplasty; THA, total hip arthroplasty; VAS, Visual Analogue Scale; and VRS, Verbal Rating Scale.

Author, Year, Country, Study Design	Study Goals	Age	N of Patients: Total (Intervention/Control)	Surgery; Groups	Type of Pain; ASA Status	Dose Regiment	Study Conclusions
Buvanendran et al., 2010 [31] USA, RCT	To determine if pregabalin has analgesic effects given preoperatively	64 (8.3), 63.3 (8.9)	240 (120/120)	TKA; pregabalin, placebo	Neuropathic pain; I, II, III	Pregabalin 300 mg orally	Perioperative pregabalin reduces the incidence of chronic neuropathic pain
Carmichael et al., 2013 [21], Canada, RCT	To assess pain (VAS, 0–10), morphine use, physical function, adverse events	18–80 pregabalin: 59.1 (10.1) placebo: 61.3 (15.1)	R: 47 (23/24) A: 31 (15/16)	THA; pregabalin, placebo	I–III	Pregabalin 75 mg twice daily, celecoxib 100 mg twice daily, 14 d preop, 2 h preop, during hospitalization, and 3 w after discharge	Pregabalin and celecoxib improves pain and physical function
Clarke et al., 2009 [5], Canada, RCT	To determine if gabapentin reduces pain and opioid use and find the efficient time of consumption	61.3 (10.7), 58.9 (9.4), 60.4 (8.1)	126 (42/42/42)	THA; gabapentin 600 mg/placebo, placebo/gabapentin 600 mg, placebo/placebo	Neuropathic pain	Gabapentin before surgery 19.06 ± 19.9 mg, after surgery 34.8 ± 13.1 mg in the first 24 h	No effect from gabapentin on morphine consumption or pain scores preoperatively/postoperatively
Clarke et al., 2015 [32], Canada, RCT	To study the effects of pregabalin on pain and functional outcomes	60.1 (8.8), 60.2 (9.5)	184 (92/92)	THA; pregabalin, placebo	Neuropathic pain; I, II, III	Pregabalin 150 mg p.o	No improvement in physical function
Jain et al., 2012 [26], India, RCT	To evaluate pain at rest and on movement (VRS, 0–10), morphine use, rescue analgesic use, patient satisfaction, sedation, adverse events	18–75 pregabalin: 59.7 (8.63) placebo: 57.1 (8.81)	40 (20/20)	TKA; pregabalin, placebo	I, II	Pregabalin 75 mg twice a day 2 h preop; 2 d postop	Pregabalin reduces opioid use, improves postop analgesia, and yields higher patient satisfaction
Martinez et al., 2014 [27], France, RCT	To assess pain at rest and on movement (NRS, 0–10), morphine use, side effects, pressure pain thresholds, secondary hyperalgesia	18–80 placebo: 64 (11) ketamine: 60 (17) pregabalin: 64 (9) ketamine + pregabalin: 59 (12)	142 (38/34/35/35) (placebo/ketamine/pregabalin/ketamine + pregabalin)	THA; placebo; ketamine; pregabalin; ketamine + pregabalin	I–III	Pregabalin 150 mg preop	The combination of pregabalin and ketamine has a small, beneficial clinical effect
Mathiesen et al., 2008 [24], Denmark, RCT	To examine morphine use, pain at rest and during mobilization (VAS, 0–100), PONV, sedation, dizziness, and ondansetron use	55–75 placebo: 66 (63–71) pregabalin: 67 (62–71) pregabalin + dexamethasone: 68 (64–71) median (range)	R: 126 (42/42/42) A: 120 (38/40/42) (placebo/pregabalin/pregabalin + dexamethasone)	THA; placebo, pregabalin, pregabalin + dexamethasone	I–III	Pregabalin 300 mg 1 h preop	Pregabalin reduced postop morphine use. This was not associated with a reduced PONV. Pregabalin resulted in increased sedation. Pregabalin and dexamethasone provided no effects on pain or opioid use
Lee et al., 2015 [9], Korea, RCT	To study the postoperative pain, analgesic drug consumption, and functional outcomes after pregabalin	Pregabalin: 63.38 (10.71), placebo: 67.60 (8.98)	87 (45/42)	TKA; pregabalin, control	Neuropathic pain; I, II, III	400 mg celecoxib plus 150 mg pregabalin—1 h prior to the operation	No difference between the two groups in functional recovery
Paul et al., 2013 [20], Canada, RCT	To assess morphine use, pain (NRS, 0–10) at rest and movement, side effects, patient satisfaction, knee ROM, hemodynamics	19–90 gabapentin: 62.1 (6.4) placebo: 63.5 (6.7)	101 (52/49)	TKA; gabapentin, placebo	I–IV	600 mg gabapentin 2 h preop; 8 h for 2 postop days	No effect on postoperative morphine consumption, pain, patient satisfaction, or length of hospital stay
Paul et al., 2015 [22], Canada, RCT	To determine if gabapentin preoperatively or postoperatively would decrease postoperative morphine consumption	60.9 (9.1), 60.5 (8.5)	102 (48/54)	THA; gabapentin, placebo	Neuropathic pain	600 mg of gabapentin	No difference between placebo in morphine consumption, side effects, or pain scores
Rasmussen et al., 2010 [25], Denmark, RCT	To assess morphine use, pain at rest and during mobilization (VAS, 0–100), PONV, sedation, dizziness, hallucination, and ondansetron use	55–85 gabapentin: 72 (68–77) placebo: 70 (67–75) median (IQR)	42 (24/18)	THA; gabapentin, placebo	I–III	1200 mg gabapentin preop	Preop gabapentin, reduced pain, but not morphine use
Singla et al., 2014 [29], USA, RCT	To assess pain (0–10), knee ROM, opioid use, safety	18–80 150 mg: 63 (8.5) 300 mg: 63.7 (8.3) placebo: 63.3 (9.5)	292 (98/96/98) (150 mg/300 mg/placebo)	TKA; pregabalin 150, pregabalin 300, placebo		150 mg pregabalin (75 mg bid) or 300 mg pregabalin (150 mg bid) 12 h and 2 h preop, 6 w postop	No significant differences between pregabalin and placebo
Tobias et al., 2019 [23], Brazil, RCT	To determine if preoperative and postoperative pregabalin is associated with a reduction in postoperative pain episodes	Pregabalin: 31 (7), placebo: 30 (7)	50 (25/25)	Knee ligament repair; pregabalin, control	Neuropathic pain; I or II	Pregabalin, 75 mg/d 7 days before and 7 days after surgery	Pregabalin decreased the consumption of analgesics with side effects of dizziness
Yadeau et al., 2015 [30], USA, RCT	To determine if postoperative pain could be reduced and to determine the side effects	66 (34–79)	120 (30/30/30/30)	TKA; placebo, pregabalin 50 mg, pregabalin 100 mg, pregabalin 150 mg	Neuropathic pain; I–III	0, 50, 100, and 150 mg pregabalin	No analgesic effect of pregabalin; side effects—reduced satisfaction with analgesia and increased drowsiness
Yik et al., 2019 [28], Singapore, RCT	To determine if pregabalin preoperatively with PCA morphine, paracetamol, and etoricoxib is effective for decreasing the morphine and if it decreases the pain scores	Pregabalin: 65.1 (50–80), placebo: 66.6 (50–83)	87 (45/42)	TKA; pregabalin, control	I, II, III	1 h before surgery: 75 mg pregabalin orally; 48 h after surgery: 75 mg dose per night	No effect on postoperative opioid dose, pain scores, or functional outcomes

**Table 2:** Cochrane risk of bias.

Study Reference	D1	D2	D3	D4	D5	Overall		
YaDeau et al., 2015 [30]	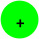	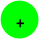	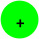	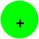	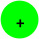	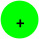	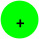	Low risk
Buvanendran et al., 2010 [31]	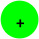	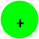	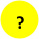	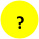	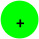	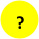	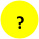	Some concerns
Clarke et al., 2009 [5]	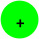	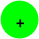	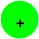	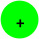	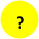	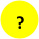	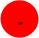	High risk
Paul et al., 2015 [22]	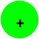	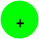	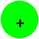	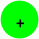	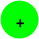	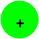		
Clarke et al., 2015 [32]	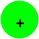	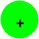	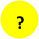	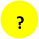	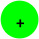	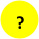	D1	Randomization process
Tobias et al., 2019 [23]	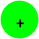	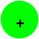	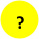	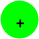	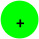	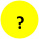	D2	Deviations from the intended interventions
Yik et al., 2019 [28]	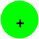	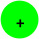	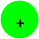	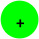	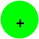	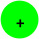	D3	Missing outcome data
Lee et al., 2015 [9]	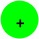	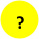	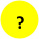	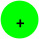	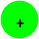	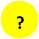	D4	Measurement of the outcome
Paul et al., 2013 [20]	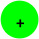	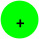	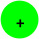	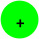	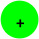	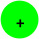	D5	Selection of the reported result
Jain et al., 2012 [26]	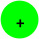	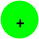	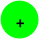	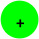	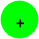	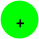		
Martinez et al., 2014 [27]	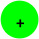	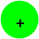	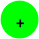	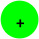	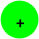	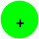		
Singla et al., 2014 [29]	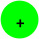	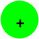	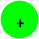	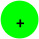	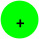	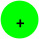		
Carmichael et al., 2014 [21]	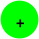	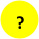	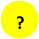	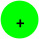	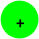	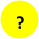		
Mathiesen et al., 2008 [24]	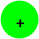	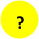	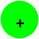	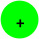	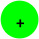	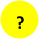		
Rasmussen et al., 2010 [25]	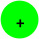	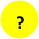	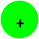	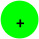	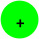	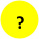		

**Table 3:** Summary of findings. Abbreviations: CI, confidence interval; GRADE, Grading of Recommendations Assessment, Development, and Evaluation; N, number; and RCT, randomized controlled trial. ⨁⨁⨁◯, moderate certainty of evidence; ⨁⨁⨁⨁, high certainty of evidence.

Outcome	Study Design	N of Patients (Studies)	Mean Difference/Relative Risk [95% CI]	Certainty of Evidence (GRADE)
Overall pain scores at rest (0–10)	RCT	1868 (11)	−0.30 [−0.55, −0.05]	⨁⨁⨁◯ Moderate
Overall pain scores on movement (0–10)	RCT	1415 (8)	−0.41 [−0.68, −0.13]	⨁⨁⨁◯ Moderate
Overall postoperative opioid use in morphine equivalent (mg)	RCT	2081 (12)	−6.42 [−9.07, −3.78]	⨁⨁⨁◯ Moderate
Overall postoperative nausea	RCT	1471 (11)	0.69 [0.55, 0.86]	⨁⨁⨁⨁ High
Overall postoperative vomiting	RCT	1173 (11)	0.72 [0.46, 1.14]	⨁⨁⨁⨁ High

## Data Availability

The datasets are available upon request from the corresponding author.
